# A rightward shift in the visuospatial attention vector with healthy aging

**DOI:** 10.3389/fnagi.2014.00113

**Published:** 2014-06-10

**Authors:** Christopher S. Y. Benwell, Gregor Thut, Ashley Grant, Monika Harvey

**Affiliations:** ^1^Centre for Cognitive Neuroimaging, School of Psychology, University of GlasgowGlasgow, UK; ^2^School of Psychology, University of GlasgowGlasgow, UK

**Keywords:** visuospatial attention, aging, landmark task, line bisection, hemispatial neglect, lateralization, spatial bias, pseudoneglect

## Abstract

The study of lateralized visuospatial attention bias in non-clinical samples has revealed a systematic group-level leftward bias (pseudoneglect), possibly as a consequence of right hemisphere (RH) dominance for visuospatial attention. Pseudoneglect appears to be modulated by age, with a reduced or even reversed bias typically present in elderly participants. It has been suggested that this shift in bias may arise due to disproportionate aging of the RH and/or an increase in complementary functional recruitment of the left hemisphere (LH) for visuospatial processing. In this study, we report rightward shifts in subjective midpoint judgment relative to healthy young participants whilst elderly participants performed a computerized version of the landmark task (in which they had to judge whether a transection mark appeared closer to the right or left end of a line) on three different line lengths. This manipulation of stimulus properties led to a similar behavioral pattern in both the young and the elderly: a rightward shift in subjective midpoint with decreasing line length, which even resulted in a systematic rightward bias in elderly participants for the shortest line length (1.98° of visual angle, VA). Overall performance precision for the task was lower in the elderly participants regardless of line length, suggesting reduced landmark task discrimination sensitivity with healthy aging. This rightward shift in the attentional vector with healthy aging is likely to result from a reduction in RH resources/dominance for attentional processing in elderly participants. The significant rightward bias in the elderly for short lines may even suggest a reversal of hemisphere dominance in favor of the LH/right visual field under specific conditions.

## Introduction

Studies of lateralized visuospatial attention in non-clinical samples have consistently revealed a slight but systematic group-level bias favoring the left visual field in young adults, a phenomenon termed “pseudoneglect” (see Voyer et al., 2012; Brooks et al., 2014 and Jewell and McCourt, 2000 for reviews). This behavioral bias is deemed to arise due to a right hemisphere (RH) dominance for visuospatial attention processing (Reuter-Lorenz et al., 1990; Fink et al., 2000a,b, 2001; Fierro et al., 2001; Foxe et al., 2003; Thiebaut de Schotten et al., 2005, 2011; Bultitude and Aimola-Davies, 2006; Ghacibeh et al., 2007; Waberski et al., 2008; Çiçek et al., 2009; Cavézian et al., 2012; Cai et al., 2013; Benwell et al., 2014) and RH dominance also seems to underlie the tendency for visuospatial neglect symptoms to occur more frequently and severely after right as compared to left hemisphere (LH) stroke (Driver and Mattingley, 1998; Vallar, 1998; Halligan et al., 2003; Harvey and Rossit, 2012). The degree of lateralized visuospatial attention bias is often assessed using variants of the horizontal line bisection task, in both clinical (Milner and Harvey, 1995; Urbanski and Bartolomeo, 2008) and non-clinical samples (Bowers and Heilman, 1980; Milner et al., 1992; Jewell and McCourt, 2000).

Though bisection performance has proven to be less consistent in older healthy adults, the systematic leftward bias appears to be attenuated, eliminated, or even reversed with age (Fukatsu et al., 1990; Stam and Bakker, 1990; Fujii et al., 1995; Jewell and McCourt, 2000; Failla et al., 2003; Goedert et al., 2010; Nagamatsu et al., 2011; Hatin et al., 2012; Loureiro et al., 2013; Brooks et al., 2014; Veronelli et al., 2014). Additionally, recent evidence suggests potential sex-differences in age-related changes in manual line bisection performance, with aging effects being strongest in males vs. relatively intact performance with aging in females (Varnava and Halligan, 2007; Barrett and Craver-Lemley, 2008; Chen et al., 2011; however see Beste et al., 2006 for discrepant results). In order to minimize the influence of motor factors on bisection decisions, Schmitz and Peigneux (2011) recently employed the Landmark Task (a non-manual, perceptual variant of line bisection) to investigate age-related changes in pseudoneglect. In this task participants are asked to estimate which of two segments of a pre-bisected line is shortest or longest (Milner et al., 1992, 1993; Harvey et al., 1995; Milner and Harvey, 1995). They found that young participants perceived the left side of equally bisected lines to be longer than the right side (typical of pseudoneglect), whereas elderly participants presented the opposite pattern, and were more accurate when unevenly bisected lines were divided on the left side. Overall, a rightward shift in the performance of older participants was found as compared to young participants, in line with previous studies (Sex of the participants was not distinguished in the study, Schmitz and Peigneux, 2011).

Several candidate models may account for the observed change in pseudoneglect with aging. One is that of Hemispheric Asymmetry Reduction in Older Adults (i.e., the HAROLD model, Cabeza, 2002). The HAROLD model suggests that functional recruitment of the non-dominant hemisphere for a given task helps to compensate for age-related unilateral working efficiency decline, resulting in reduced asymmetry in processing for the task at hand (Cabeza, 2002; Reuter-Lorenz and Cappell, 2008; Li et al., 2009). The HAROLD model has largely been investigated in the context of memory tasks and its predictions have often been supported (Bäckman et al., 1997; Grady et al., 2002; Logan et al., 2002; Cabeza et al., 2004; Rossi et al., 2004; Solé-Padullés et al., 2006; Schmitz et al., 2013). Using positron emission tomography (PET), Reuter-Lorenz et al. (2000) found prefrontal cortex (PFC) activity to be lateralized to the respective dominant hemisphere for a given stimulus in young participants. However, in elderly participants the activity was bilateral for all stimulus types. Although mainly observed in the PFC, the HAROLD model may also apply to other regions and tasks (Collins and Mohr, 2013). Nielson et al. (2002) found that during an inhibition task, parietal activity was right lateralized in young participants yet bilateral in older participants. Thus in the context of visuospatial attention biases, when performing the landmark task, elderly participants may recruit supplementary contralateral (left) brain areas in a compensatory manner, resulting in the observed absence or reversal of pseudoneglect.

Another model emphasizes accelerated aging in the right relative to the LH (Brown and Jaffe, 1975; Goldstein and Shelly, 1981), which may in turn reduce the functional dominance of visuospatial attention processing in the RH. Using a test battery designed to diagnose lateralized brain injury, it has previously been found that the performance of elderly participants is analogous to that of RH damaged patients (Klisz, 1978) and more recently specific RH impairment in elderly participants has been found during performance of a variety of psychophysical tasks (Jenkins et al., 2000; Lux et al., 2008; Nagamatsu et al., 2011; Chokron et al., 2013). The absence or reversal of pseudoneglect presented by elderly participants may therefore reflect general RH decline. However, evidence supporting greater aging of the RH in comparison to the left has been mixed (Dolcos et al., 2002; Sowell et al., 2003; Raz et al., 2004).

Additionally, rightward spatial biases are often associated with states of both tonic and chronic reduced arousal (Bellgrove et al., 2004; Manly et al., 2005; Fimm et al., 2006; Dufour et al., 2007; Dodds et al., 2008; Heber et al., 2008; Matthias et al., 2010; Benwell et al., 2013a,b; Newman et al., 2013). It is possible that a reduction in general alertness/vigilance over the lifespan (Robinson and Kertzman, 1990; Buysse et al., 2005; Nebes et al., 2009; Goedert et al., 2010) may also contribute to the chronic attenuation of pseudoneglect in the elderly.

Interestingly, the degree of visuospatial bias displayed during landmark task performance is modulated within participants by stimulus properties such as line length. Recent studies employing the landmark task in healthy young participants have shown that while long lines (subtending >6° horizontal visual angle (VA) in length) induce a systematic (usually left) bias, short lines (subtending <2° VA) induce either no bias or a right bias (McCourt and Jewell, 1999; Rueckert et al., 2002; Rueckert and McFadden, 2004; Heber et al., 2010; Thomas et al., 2012; Benwell et al., 2013a, 2014). The line length effect appears to arise due to asymmetrical hemispheric contributions (in favor of the RH) to the perceived salience of line stimuli that is more pronounced for long than short lines and hence a left bias arises more prominently for long lines (Anderson, 1996; Benwell et al., 2014). In a recent study, we manipulated both time-on-task/vigilance and line length in a sample of healthy young participants (Benwell et al., 2013a). We found the rightward shifting effects of time-on-task and line length to be additive: at baseline the common group-level leftward bias was observed in long lines whereas no systematic bias was observed in short lines (group average not significantly different from veridical center). After 1 h of prolonged performance of the landmark task with long lines, both long and short line performance were tested again. A rightward shift in bias was evident in that the left bias was now absent in long lines, and intriguingly the rightward shift also transferred to the un-practiced short lines which now evidenced a right bias significantly different from veridical center. The additive effects of reduced line length and increased time-on-task suggest that both manipulations may result in down-regulation of RH attention network engagement and hence the observed rightward shifts in spatial bias. Additionally, an overall task performance decrement (as indexed by the curve width of the fitted psychometric function) was observed with prolonged time-on-task, further suggesting a degradation of attentional resources.

Elucidating how the established bias modulators of age and line length interact to influence lateralized visuospatial bias as displayed during landmark task performance will allow for a refinement of models of visual attention processing changes with healthy aging. To investigate this, we compared landmark task performance on three different line lengths (short, medium and long) between young and elderly healthy participants. In line with previous studies, we predicted a systematic leftward bias for long lines in young participants that would be attenuated with reducing line length. If hemispheric asymmetry reduction alone accounts for the attenuation of pseudoneglect with aging then we would expect to see no systematic bias for any line length in the elderly and also relatively preserved overall performance on the task. Alternatively, if reduced RH function and/or chronic reduced arousal play a role in the attenuation of bias then we would expect to see a pattern of performance in the elderly analogous to that previously observed in young participants following prolonged time-on-task: namely no bias in long lines and a systematic rightward bias for short lines along with an overall task performance decrement (Benwell et al., 2013a).

## Material and methods

### Participants

Twenty right-handed young (12 males, mean age = 23.25 years; SD = 2.83, max = 31, min = 18) and 20 right-handed elderly participants (11 males, mean age = 68.45 years; SD = 4.95, max = 77, min = 60) took part in the experiment. Written informed consent was obtained from each participant. All participants were volunteers naive to the experimental hypothesis being tested. All participants had normal or corrected-to-normal vision and reported no history of neurological disorder. The experiment was carried out within the Institute of Neuroscience and Psychology at the University of Glasgow and was approved by the local ethics committee.

### Instrumentation and stimuli

**Figure 1:**
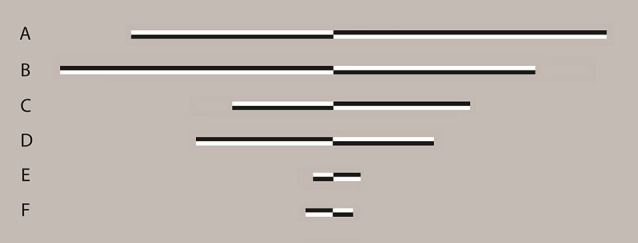
**Examples of line stimuli used in the experiment** Lines were transected at 1 of 13 locations ranging symmetrically from ±7.5% (distance between transector locations = 1.25%) of absolute line length relative to and including veridical center. In long lines, this represented a range of −1.48° to 1.48° of visual angle (VA) with a distance between transector locations of 0.25° of VA. In medium lines a range of −0.74° to 0.74° of VA with a distance between transector locations of 0.12° of VA was presented and in short lines, a range of −0.15° to 0.15° of VA with a distance between transector locations of 0.02° of VA was presented. All lines were displayed with the transector location centered on the vertical midline of the display (i.e., aligned to a central fixation cross which preceded the presentation of the lines). Lines A, C and E are transected to the left of veridical center whereas lines B, D and F are transected to the right of veridical center. Lines of varying contrast polarity appeared with equal frequency and the order of appearance was randomized.

Stimuli were presented using the E-Prime software package (Schneider et al., 2002) on a CRT monitor with a 1280 × 1024 pixel resolution and 85 Hz refresh rate. Adapted from experiment 2 of Benwell et al. (2013b), the paradigm represented a computerized version of the landmark task (Milner et al., 1992; McCourt and Olafson, 1997; Olk and Harvey, 2002). Lines of 100% Michelson contrast were presented on a gray background (luminance = 179, hue = 179). Figure 1 shows examples of line stimuli used in the experiment. Three different lengths of line were presented. “Long” lines measured 24.3 cm in length by 0.5 cm in height and at a viewing distance of 70 cm subtended 19.67° (width) by 0.4° (height) of VA. At the same viewing distance, “medium” lines measuring 12.15 cm × 0.5 cm subtended 9.92° × 0.4° of VA and “short” lines measuring 2.43 cm × 0.5 cm subtended 1.98° by 0.4° of VA.

All three line lengths were transected at 1 of 13 points ranging from ±7.5% (distance between transector locations = 1.25%) of absolute line length to veridical center. In long lines, this represented a range of −1.48° to 1.48° of VA with a distance between transector locations of 0.25° of VA. In medium lines a range of −0.74° to 0.74° of VA with a distance between transector locations of 0.12° of VA was presented and in short lines, a range of −0.15° to 0.15° of VA with a distance between transector locations of 0.02° of VA was presented. All lines were displayed with the transector location centered on the vertical midline of the display (i.e., aligned to a central fixation cross which preceded the presentation of the lines, see below).

### Procedure

Participants were seated with their midsagittal plane aligned with the display monitor. Viewing distance (70 cm) was kept constant using a chin rest. Each trial began with presentation of a fixation cross (0.4° (height) × 0.4° (width) of VA) for 1 s followed by presentation of the transected line (150 ms). The transection mark was always aligned with the fixation cross (i.e., the eccentricity of the line endpoints varied across trials while the transection point always appeared at the same central position), therefore preventing use of the fixation cross as a reference point for bisection judgments. The fixation cross then reappeared for the duration of the response period during which participants indicated which end of the line had appeared longest/shortest to them by pressing either the left or right response key. Half of the participants were asked to judge which end of each line was longest and the other half were asked to judge which end was shortest, in order to prevent any possible group-level response bias (increased likelihood of pressing either the left or right response key regardless of the visual percept, especially in cases of uncertainty (see Morgan et al., 2012; García-Pérez and Alcalá-Quintana, 2013)) from contaminating the perceptual midpoint analysis.

**Figure 2:**
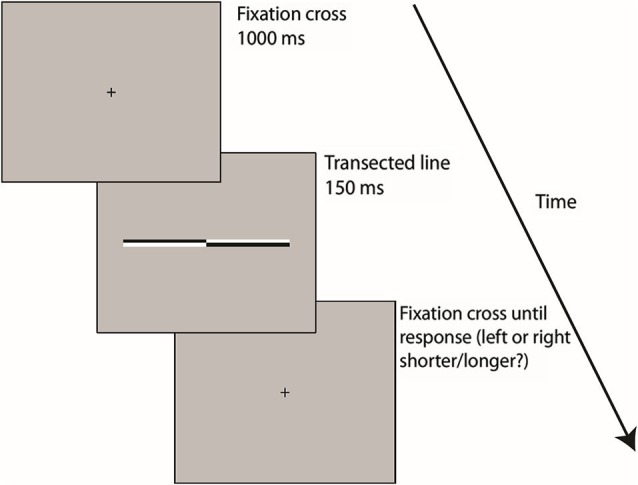
**A schematic representation of the trial procedure** Following 1000 ms presentation of a fixation cross, transected lines were presented for 150 ms before reappearance of the fixation cross on the screen until the subject responded, by pressing either the left or right (shorter/longer?) response key. The subsequent trial began as soon as the response was made.

Participants always responded using their dominant right hand (right index and middle finger respectively) and were instructed to hold their gaze on the center of the screen throughout each trial. The subsequent trial began as soon as the response was made. Trials lasted approximately 2 s. Trial type (location of transector in line) was selected at random. Each participant completed 91 trials of each line length (Overall = 273 trials, 7 judgments at each of the 13 transector locations) split into 7 short blocks (lasting approximately 2–3 min). Participants were allowed to take as long a break as they wished between blocks. A block of 20 practice trials was performed immediately prior to the beginning of the experimental blocks. The entire experiment lasted approximately 20–25 min (see Figure 2 for schematic representation of the trial procedure).

### Analysis

In order to obtain an objective measure of perceived line midpoint for each line length in each subject, psychometric functions (PFs) were derived using the method of constant stimuli. The dependent measure was the proportion of trials on which participants indicated that the transector had appeared closer to the left end of the line. Non-linear least-squares regression was used to fit a cumulative logistic function to the data for each line length in each subject. The cumulative logistic function is described by the equation:

f(μ,x,s)=1/(1+exp((x−μ)/s)))

where *x* are the tested transector locations, *μ* corresponds to the *x*-axis location with a 50% “left” and 50% “right” response rate and *s* indexes the width of the nonasymptotic region of the fitted curve. The 50% location is known as the point of subjective equality point of subjective equality (PSE) and represents an objective measure of perceived line midpoint. The width of the PF provides a measure of the precision of participants’ line midpoint judgments per line length. A low width value indicates that the PF is steep and that the observer can discriminate differences between transector locations relatively easily, whereas a high width value indicates that the PF is shallow and that the observer can only discriminate relatively coarse differences (Fründ et al., 2011). Inferential statistical analyses were performed on the individually fitted PF PSE and width estimates.

## Results

### Subjective midpoint (P.S.E) analysis

**Figure 3:**
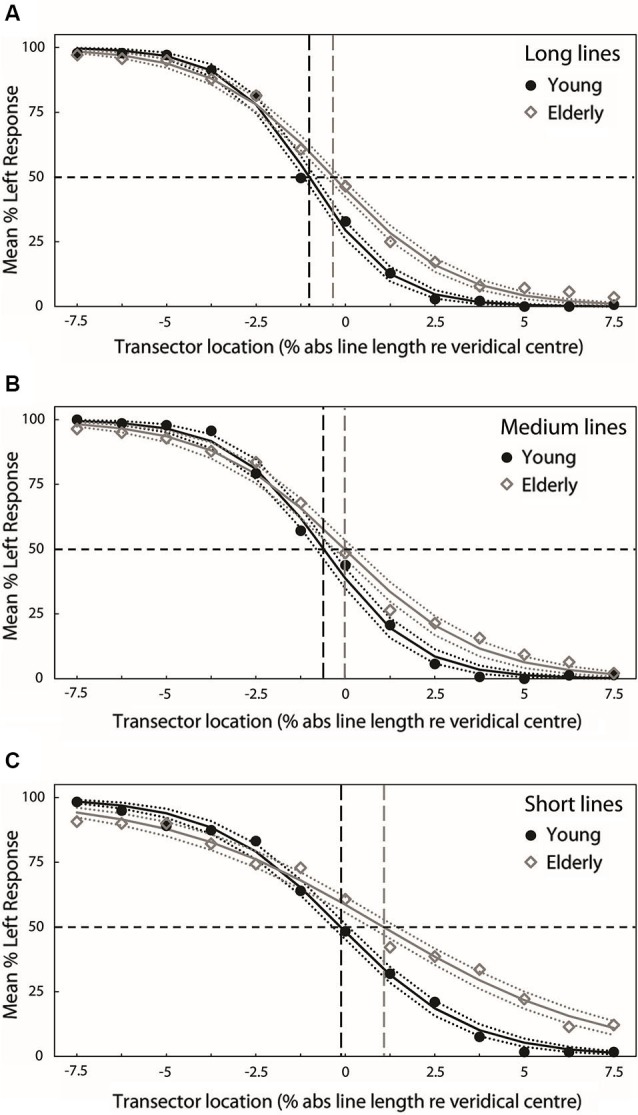
**Group-averaged PFs for both experimental groups at each line length (A = long, B = medium, C = short)**. For each line length, black filled circle symbols (young participants) and gray open diamond symbols (elderly participants) plot mean percentage left response as a function of transector location. The black (young) and gray (elderly) smooth curves represent the best-fitting least-squares cumulative logistic PFs (95% confidence interval represented by black (young) and gray (elderly) dotted lines). Where black (young) and gray (elderly) vertical dashed lines cross the black horizontal dashed lines indicate the transector locations corresponding to the 50% response rates (PSEs).

Figures 3A–C present group-averaged PFs for both experimental groups at each line length. For each line length, black filled circle symbols (young participants) and gray open diamond symbols (elderly participants) plot mean percentage left response as a function of transector location. The black (young) and gray (elderly) smooth curves represent the best-fitting least-squares cumulative logistic PFs (95% confidence interval represented by black (young) and gray (elderly) dotted lines). Where black (young) and gray (elderly) vertical dashed lines cross the black horizontal dashed lines indicate the transector locations corresponding to the 50% response rates (PSEs).

**Figure 4:**
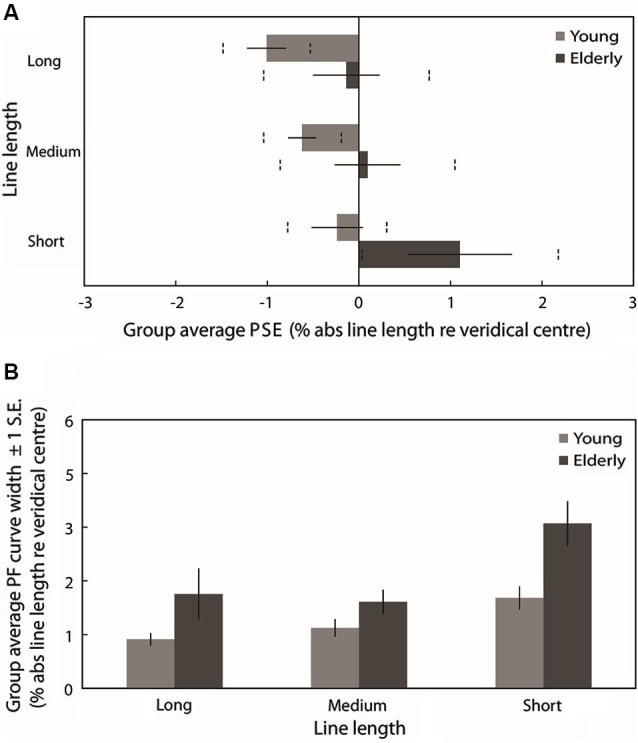
**(A)** Group averaged PSE values (±1 standard error (S.E.), vertical dashed lines represent 95% confidence intervals (CIs)) obtained from PFs fitted to the individual participants’ data for each line length. Light gray bars represent the young group and dark gray bars represent the elderly group. **(B)** Group averaged PF curve width (±1 S.E.) obtained from PFs fitted to the individual participants’ data for each line length.

Figure 4A plots the group mean PSEs (±1 standard error (S.E.), vertical dashed lines represent 95% confidence intervals (CIs)) obtained from PFs fitted to the individual participants’ data for each line length. These are in close agreement with the group averaged PF PSEs. In line with previous studies of pseudoneglect, mean long line PSE in the young group was displaced to the left of veridical center by −1% of absolute line length and this leftward bias was significantly different from veridical center (95% CI does not include 0) whereas in the elderly group the mean PSE was slightly to the left (−0.14%) but not significantly different from veridical center (95% includes 0). Mean medium line PSE in the young group was displaced to the left of veridical center by −0.62% and this leftward bias was also significantly different from veridical center (95% CI does not include 0). In contrast, the medium line elderly PSE was very slightly to the right of center by 0.1% but again not significantly different from veridical center (95% CI includes 0). In the short lines, mean PSE in the young group was −0.24% to the left of veridical center but the difference from veridical center was not significant (95% CI includes 0) whereas mean PSE in the elderly group was significantly displaced to the right of veridical center by 1.1% (95% CI does not include 0).

A 2 (Age group: young vs. elderly) × 3 (Line length: long vs. medium vs. short) ANOVA on individually fitted PF PSEs revealed a significant main effect of age group (*F*_(1,38)_ = 5.830, *p* = 0.021, $\eta_{p}^{2}=0.133$), a significant main effect of line length (*F*_(2,76)_ = 6.509, *p* = 0.002, $\eta_{p}^{2}=0.146$) but no significant age group × line length interaction (*F*_(2,76)_ = 0.524, *p* = 0.524, $\eta_{p}^{2}=0.017$). The overall subjective midpoint was significantly more to the left in the young participants than in the elderly (as indexed by the PSEs), indicating a group level rightward shift in the attentional vector with age (as is clearly displayed in Figure 4A). Pairwise comparisons (Bonferroni-corrected) to analyze the simple effects of line length revealed no statistically significant difference in subjective midpoint between either long and medium lines (*t*_(39)_ = −1.846, *p* = 0.226, Cohen’s *d* = −0.292) or medium and short lines (*t*_(39)_ = −2.163, *p* = 0.111, Cohen’s *d* = −0.345) but a significant rightward shift in subjective midpoint from long to short lines (*t*_(39)_ = −3.022, *p* = 0.014, Cohen’s *d* = −0.482) regardless of age (again displayed in Figure 4A). Additionally, a within-subjects linear contrast analysis revealed a significant linear shift in bias with line length (*F*_(1,38)_ = 9.017, *p* = 0.005, $\eta_{p}^{2}=0.192$).

### Psychometric function curve width analysis

Figure 4B plots the mean PF curve width (±1 S.E.) obtained from PFs fitted to the individual participants’ data for each line length. A 2 (Age group: young vs. elderly) × 3 (Line length: long vs. medium vs. short) ANOVA revealed a significant main effect of age group (*F*_(1,38)_ = 8.674, *p* = 0.005, $\eta_{p}^{2}=0.186$), a significant main effect of line length (*F*_(2,76)_ = 11.637, *p* < 0.001, $\eta_{p}^{2}=0.234$) and no significant age group × line length interaction (*F*_(2,76)_ = 1.706, *p* = 0.188, $\eta_{p}^{2}=0.043$). PF curve widths were significantly shallower in elderly participants than in young participants, indicating reduced discrimination sensitivity with age. Pairwise comparisons (Bonferroni-corrected) to analyze the simple effects of line length revealed no statistically significant difference in PF width between long and medium lines (*t*_(39)_ = −0.155, *p* = 1, Cohen’s *d* = −0.033) but a significant increase in width from both long to short lines (*t*_(39)_ = −3.409, *p* = 0.005, Cohen’s *d* = −0.542) and from medium to short lines (*t*_(39)_ = −4.845, *p* < 0.001, Cohen’s *d* = −0.881). A within-subjects linear contrast analysis revealed a significant linear shift in curve width with line length (*F*_(1,38)_ = 11.56, *p* = 0.002, $\eta_{p}^{2}=0.233$). Discrimination sensitivity for the task was significantly lower for short lines than for long and medium lines regardless of age (as displayed in Figure 4B).

### Additional gender analysis

Recent evidence from studies employing manual line bisection has suggested potential sex-differences in age-related changes in bisection performance, with aging effects being strongest in males vs. relatively intact performance with aging in females (Varnava and Halligan, 2007; Barrett and Craver-Lemley, 2008; Chen et al., 2011; however see Beste et al., 2006 for discrepant results). In order to test for any such gender effects in age-related changes in landmark task performance, we re-analyzed *(post hoc)* the PSE and width values with an additional between-subjects factor of gender (female, male) included in the ANOVAs. The PSE re-analysis revealed no additional main effect of gender (*F*_(1,36)_= 0.019, *p* = 0.892, $\eta_{p}^{2}=0.001$) and no significant interaction between either age group × gender (*F*_(1,36)_= 0.411, *p* = 0.525, $\eta_{p}^{2}=0.011$), length × gender (*F*_(2,72)_ = 0.337, *p* = 0.715, $\eta_{p}^{2}=0.009$) nor age group × length × gender (*F*_(2,72)_ = 0.608, *p* = 0.547, $\eta_{p}^{2}=0.017$).

The width re-analysis also revealed no main effect of gender (*F*_(1,36)_= 0.970, *p* = 0.331, $\eta_{p}^{2}=0.026$), no significant interaction between either age group × gender (*F*_(1,36)_= 0.299, *p* = 0.588, $\eta_{p}^{2}=0.008$), length × gender (*F*_(2,72)_ = 0.615, *p* = 0.543, $\eta_{p}^{2}=0.017$) nor age group × length × gender (*F*_(2,72)_ = 0.958, *p* = 0.388, $\eta_{p}^{2}=0.026$).

## Discussion

Recent studies have shown age-related changes in the expression of visual pseudoneglect (Fukatsu et al., 1990; Stam and Bakker, 1990; Fujii et al., 1995; Jewell and McCourt, 2000; Failla et al., 2003; Barrett and Craver-Lemley, 2008; Goedert et al., 2010; Nagamatsu et al., 2011; Schmitz and Peigneux, 2011; Hatin et al., 2012; Loureiro et al., 2013; Veronelli et al., 2014). We aimed to investigate, for the first time, how the established line bisection bias modulator of line length interacts with healthy aging to influence lateralized visuospatial bias as displayed during landmark task performance. For this purpose, we compared landmark task performance on three different line lengths (short, medium and long) between young (18–31 years old) and elderly (60–77) healthy participants.

As expected, young participants displayed a group-level systematic leftward bias (pseudoneglect) during long line landmark task performance. This leftward bias was reduced for the medium length lines and no systematic bias was observed for performance of the task with short lines, confirming the previously reported line-length effect (McCourt and Jewell, 1999; Rueckert et al., 2002; Rueckert and McFadden, 2004; Heber et al., 2010; Thomas et al., 2012; Benwell et al., 2013a, 2014). Moreover, the results revealed a group-level rightward shift in the visuospatial attention vector in the elderly as compared to the young participants, in line with previous findings of an attenuation or reversal of pseudoneglect with healthy aging (Fukatsu et al., 1990; Stam and Bakker, 1990; Fujii et al., 1995; Jewell and McCourt, 2000; Failla et al., 2003; Barrett and Craver-Lemley, 2008; Goedert et al., 2010; Nagamatsu et al., 2011; Schmitz and Peigneux, 2011; Hatin et al., 2012; Loureiro et al., 2013; Veronelli et al., 2014). Importantly, no interaction was observed between age group and line length suggesting that the elderly participants were subject to the line length effect in a similar manner to the young (i.e., a rightward shift in subjective midpoint with reduced line length). We found no effect of gender on landmark task performance in either the young or the elderly.

Our results replicate and extend those of Schmitz and Peigneux (2011) who found suppression, and near reversal, of the leftward pseudoneglect bias in their elderly sample during long line landmark performance. In their study, the line stimuli remained onscreen until the participant responded (free-viewing). The authors note that this absence of control of ocular scanning in their study precluded them from dissociating a true perceptual bias shift with aging from a failure of inhibition of return (IOR). IOR represents a mechanism by which the viewer disengages from previously processed aspects of a stimulus in order to facilitate perception of its entirety (Posner and Cohen, 1984). Using a stimulus duration of 150 ms only (and thus preventing eye movements), we here confirm that the observed rightward shift in the attention vector with healthy aging is unlikely to occur as a result of a failure of IOR.

### Potential neural mechanisms of the rightward perceptual shift with aging

#### Accelerated right hemisphere aging/HAROLD model

Previous studies exploring age-related variability in neurocognitive function have posited a decline in hemispheric specialization of task-related neural activity to represent a form of compensation for age-related deficits that supports task performance (Reuter-Lorenz and Lustig, 2005; Reuter-Lorenz and Cappell, 2008; Angel et al., 2011). However, the functional significance of the observed neural activation of regions not primarily associated with task performance in young participants, and whether such “recruitment” is restricted to elderly participants, remains unclear (Reuter-Lorenz and Park, 2010; Friedman, 2013).

Though the rightward shift in the visual attention vector with age observed in the current study would support an increased involvement of the LH in task processing in the elderly compared to the young participants (Cabeza, 2002; Reuter-Lorenz and Cappell, 2008; Li et al., 2009), the HAROLD model alone appears to be inconsistent with the findings of a significant rightward bias for short lines in the elderly in the current study along with previous reports of group-level rightward bisection biases in elderly samples (Stam and Bakker, 1990; Fujii et al., 1995). The HAROLD model would predict symmetrical bisection behavior in elderly participants but it would not predict systematic right biases beyond the veridical midline (Brooks et al., 2014). Additionally, we found overall performance precision (as indexed by the curve width of the fitted psychometric functions) to be lower in elderly participants suggesting reduced discrimination sensitivity with aging. Although the influence of low level visual deficits (such as reduced visual resolution) cannot be ruled out, elderly participants were less able to successfully discriminate between the different transector locations (for all three line lengths) and so “compensatory” recruitment of the LH for landmark task processing does not equate to preserved task performance ability equivalent to that of young participants.

Moreover, increased LH involvement could occur as a result of reduced inhibitory influence of the RH, in line with an interhemispheric competition account of spatial attention control (Kinsbourne, 1977; Duecker et al., 2013; Szczepanski and Kastner, 2013) in combination with accelerated RH aging (Brown and Jaffe, 1975; Goldstein and Shelly, 1981; Nagamatsu et al., 2011) and/or a decline in corpus callosum integrity with age (Hausmann et al., 2003; Sullivan and Pfefferbaum, 2006; Koch et al., 2007, 2011).

#### Potential role of arousal and/or perceptual load

Rightward spatial biases are often associated with states of both tonic and chronic reduced arousal (Bellgrove et al., 2004; Manly et al., 2005; Fimm et al., 2006; Dufour et al., 2007; Dodds et al., 2008; Heber et al., 2008; Matthias et al., 2010; Newman et al., 2013; Benwell et al., 2013a,b). In fact, after 1 h of landmark task performance with long lines, a rightward shift in the attentional vector was displayed by the young participants in our previous study (including a rightward bias for short lines that was significantly different from veridical center) (Benwell et al., 2013a). This pattern of bisection behavior was remarkably similar to that displayed at baseline by the elderly sample in the current study. It is possible that a reduction in general alertness over the lifespan (Robinson and Kertzman, 1990; Goedert et al., 2010; Buysse et al., 2005; Nebes et al., 2009), and/or a reduction in functional interaction between RH ventral and dorsal networks subserving visuospatial attention (see Thiebaut de Schotten et al., 2011 and the discussion of Benwell et al., 2013b), may contribute to a chronic attenuation of pseudoneglect in aged individuals. Additionally, the increased difficulty of performing the task with short lines (as indexed by the shallower PF curve width values) may further hinder RH contribution to the task in states of sub-optimal function (such as with aging (Brown and Jaffe, 1975; Goldstein and Shelly, 1981; Nagamatsu et al., 2011) or reduced vigilance/increased time-on-task (Fimm et al., 2006; Benwell et al., 2013a,b)) and hence bring about the observed rightward biases.

### Line length effect and aging

#### Potential neural mechanisms

The current results show for the first time that, despite an overall rightward shift in midpoint judgments in the elderly, reducing line length results in the same pattern of behavior in the elderly as in the young (i.e., a rightward shift in subjective midpoint) during landmark task performance. The rightward shifting effects of age and line length on midpoint judgment appear to be additive. In a mathematical model of bisection behavior, the line length effect was posited to arise due to asymmetrical hemispheric contributions (in favor of the RH) to the perceived salience of line stimuli that is more pronounced for long than short lines (Anderson, 1996). We have recently investigated the neural correlates of the line length effect in neurologically normal young participants during performance of the landmark task (Benwell et al., 2014). Our EEG results showed that increased engagement of regions of the right lateralized ventral attention network in long relative to short lines contributes to the genesis of the spatial bias: we found an ERP response which showed higher amplitude to long as compared to short lines, corresponded in its timing to the N1-component and was right lateralized to areas of the temporo-parietal junction (TPJ; Benwell et al., 2014). Furthermore, the difference in peak N1-amplitude between long and short line processing correlated with the difference in line bisection bias between long and short lines across participants, thereby providing empirical support for Anderson’s (1996) model. The TPJ represents a key node in the ventral frontoparietal attention network implicated in both the orienting of visuospatial attention and the maintenance of arousal (Corbetta and Shulman, 2002, 2011). De-regulation of RH TPJ activity is thought in turn to reduce activation of the bihemispheric dorsal frontoparietal network (implicated in the distribution of visuospatial attention across the visual field) and has been linked to rightward shifts in visuospatial bias in healthy participants (O’Connell et al., 2011; Newman et al., 2013; Benwell et al., 2013b). We posit that these neural correlates may also underlie the length effect observed here in the elderly, over and above any age-related changes in task processing.

#### No evidence for gender specific effects

Varnava and Halligan (2007) employed manual line bisection to investigate the effects of age and gender on bisection performance in healthy participants on three different line lengths comparable to those used in the current study. In their study, only males showed a rightward shift in bisection bias with age and only for long line performance. This effect of gender on manual line bisection performance with aging has been supported by subsequent studies, with the effect of aging appearing to be strongest for males (Barrett and Craver-Lemley, 2008; Chen et al., 2011). A possible explanation for the discrepant finding of no sex difference in the current study could be the use of the landmark task instead of manual line bisection (Varnava and Halligan, 2007; Barrett and Craver-Lemley, 2008; Chen et al., 2011). In general, differences in experimental procedure (such as the viewing distance employed (see McCourt and Garlinghouse, 2000; Varnava et al., 2002; Longo and Lourenco, 2006)), sample demographics and analysis techniques across studies may contribute to the differential findings. Treating age as a continuous variable in a sample of participants largely over 40 years old (mean age = 58.7, only 5 out of 44 participants <40), Chen et al. (2011) dissociated “where” perceptual errors from “aiming” motor errors during line bisection and found a rightward shift in perceptual midpoint with aging in men only. Thus, further research should aim to explore, ideally in larger samples and utilizing the deployment of multiple visuospatial tasks and analysis techniques, the reasons underlying these discrepancies in gender- and age-related effects on visuospatial bias. Although the current experiment was not explicitly set up to investigate gender differences, we would propose that non-perceptual factors may contribute to the previously observed gender specific aging effects in pseudoneglect, and that both sexes appear to experience a rightward perceptual shift in the visuospatial attention vector with healthy aging.

#### Comparison to neglect

The pattern of the line length effect displayed by our elderly sample is in the opposite direction to that often observed in unilateral neglect patients. In these patients, a reduction in line length generally results in a systematic reduction of the severe rightward bias typically exhibited on long lines, with a leftward bias sometimes being displayed on very short lines (the “crossover” effect Halligan and Marshall, 1988; Marshall and Halligan, 1989; Harvey et al., 1995; Anderson, 1996, 1997; Monaghan and Shillcock, 1998, 2004; Ricci and Chatterjee, 2001; Mennemeier et al., 2005; Veronelli et al., 2014). We therefore think it unlikely that the performance of elderly participants can be seen as a mild version of spatial neglect. What seems to be the case is that the elderly participants show an overall rightward shift in the attentional vector, that is most pronounced for the short lines. However, the comparison of findings from healthy participants with those in neglect patients and the “crossover” literature is complicated by the large variance of line bisection performance patterns both within and across patients (Halligan et al., 1990) and common concurrent primary visual and motor deficits post-stroke (Doricchi et al., 2005; Binetti et al., 2011; Kerkhoff and Schenk, 2011). The 150 ms landmark task presentation duration employed here minimizes the influence of non-perceptual motor components such as hand use and visual scanning on bisection decisions (Milner et al., 1992; Luh, 1995; Bisiach et al., 1998; Toraldo et al., 2004). Employing the paradigm from the current study in RH stroke neglect patients both with and without concomitant primary visual deficits would be highly informative in terms of elucidating further purely perceptual contributions to the line length effect in neglect and the potential role played by primary visual deficits in the commonly observed “crossover” effect (Doricchi et al., 2005; Binetti et al., 2011).

### Future directions

The neural origin(s) of the additive effects of aging and line length remain unclear. It is possible that two independent processes influencing spatial bias are at play, one affected by aging (leading to a rightward shift) and the other unaffected (preserving the line length effect in healthy aging). The introduction of neuroimaging techniques is likely to represent an important step with regard to answering this and many more of the open questions pertaining to visuospatial processing in the elderly. To our knowledge, neuroimaging studies of bisection task performance to date have been restricted to young healthy participants, revealing strong RH dominance for task processing (Fink et al., 2000a,b, 2001; Foxe et al., 2003; Waberski et al., 2008; Çiçek et al., 2009; Thiebaut de Schotten et al., 2011; Cavézian et al., 2012; Benwell et al., 2014). Using EEG and a passive viewing task, De Sanctis et al. (2008) showed reduced hemispheric asymmetry of early-visual processing in elderly compared to young participants. As mentioned, we have linked the genesis of the landmark task bias to the RH amplitude of an early component (N1) of the visual evoked potential (Benwell et al., 2014). In addition, the magnitude and direction of bias have also been linked to the relative anatomical hemispheric lateralization of a parieto-frontal white matter pathway (Thiebaut de Schotten et al., 2011). Investigation of these neural modulators of visuospatial bias in the elderly represents a natural and potentially illuminating next step.

## Author contributions

Christopher S.Y. Benwell conceived the experiment, analyzed the data and co-wrote the manuscript. Monika Harvey and Gregor Thut supervised the entire work and co-wrote the manuscript. Ashley Grant collected and analyzed the data.

## Conflict of interest statement

The authors declare that the research was conducted in the absence of any commercial or financial relationships that could be construed as a potential conflict of interest.
